# Promoting Employee Organizational Citizenship Behavior (OCB) in Small- and Medium-Sized Enterprises: A Cognitive and Affective Perspective on Ethical Leadership

**DOI:** 10.3390/bs15030380

**Published:** 2025-03-18

**Authors:** Wei Su, Juhee Hahn

**Affiliations:** 1 School of Management, Putian University, Putian 351100, China; suv4591@gmail.com; 2 Department of Business Management, Chung-Ang University, Seoul 06974, Republic of Korea

**Keywords:** ethical leadership, team ethical climate, ethical role modeling, affective well-being, organizational citizenship behavior, SMEs

## Abstract

Compared to the formal rules and regulations of large companies, leadership behavior has a greater influence on employee behaviors in small and medium enterprises (SMEs). Unlike large enterprises, many SMEs have a weaker market position, and their survival and development depend on employees’ willingness to make additional efforts beyond their formal job duties. Thus, this study focuses on SME employees to explore the effect of ethical leadership on subordinates’ organizational citizenship behavior (OCB). This study proposes a multilevel mediating model, where ethical climate and ethical role modeling represent cognitive social learning perspectives at the team and individual levels, respectively, while affective well-being serves as an individual-level affective perspective. A total of 426 valid questionnaires from 71 teams were collected, and MPLUS was used to verify the study hypotheses. The results indicate that (1) ethical leadership has a significant positive impact on employee OCB; (2) ethical leadership also significantly affects team ethical climate, ethical role modeling, and affective well-being; and (3) the partial mediating effects of team ethical climate, ethical role modeling, and affective well-being are confirmed. This research provides empirical evidence for the mechanism between ethical leadership and employee OCB in SMEs.

## 1. Introduction

Since many SMEs are in a weaker market position than large enterprises, their survival and development heavily rely on employees’ willingness to make additional efforts beyond their formal job duties (Popescu et al., 2015). Employee organizational citizenship behavior (OCB) serves as a key mechanism for achieving it. However, prosocial and voluntary employee OCB (Hart & Hart, 2023) does not always naturally occur as often as expected. Scholars have examined various factors that drive OCB, among which ethical leadership is important (Ng & Feldman, 2015). However, most leadership research has focused on large organizations, with relatively little attention given to SMEs (Madanchian et al., 2018). This gap persists despite evidence that, in SMEs, leaders’ behavior has a greater influence on employee behaviors than formal rules and regulations do in large companies (Kararti & Yuksekbilgili, 2014). Therefore, this study aims to bridge this gap by exploring the connection between ethical leadership and subordinates’ organizational citizenship behavior in SMEs.

Brown et al. (2005) identified two key social learning pillars of ethical leadership: the moral person and the moral manager. However, few studies have empirically examined social learning as a mechanism for explaining the connection between ethical leadership and employee OCB (Bai et al., 2019). To address this gap, this study empirically examines the relationship between ethical leadership and employee OCB from these two social learning perspectives. Moreover, extant studies have yet to combine the ethical climate (as a group-level ethical leadership mechanism) with ethical role modeling (as an individual-level ethical leadership mechanism). Thus, this study proposes a multilevel social learning model for ethical leadership, integrating ethical role modeling (at the individual level) and ethical climate (at the group level).

The significance of employee well-being is being increasingly emphasized due to the rise of positive psychology and positive organizational behavior (Li et al., 2014). Employees’ affective well-being is essential to organizations, as it is positively associated with creativity (Lin et al., 2014) and job performance (Khoreva & Wechtler, 2018) while being negatively related to turnover intention (Chang et al., 2017). However, limited research has explored the relationship between affective well-being and OCB, a key form of extra-role performance. Further investigation is needed to understand how and why affective well-being affects employees’ OCB (Xu et al., 2019). Therefore, beyond ethical climate and role modeling at the cognitive level, this study incorporates affective well-being as an emotional factor to analyze the mechanism linking ethical leadership and organizational citizenship behavior.

This study contributes significantly to the literature by proposing a multilevel social learning model that conceptualizes ethical leadership mechanisms at both the individual level (moral person–ethical role modeling) and the group level (moral manager–ethical climate). In addition to the cognitive perspective of social learning, this study incorporates affective well-being from positive psychology to examine ethical leadership mechanisms from an affective perspective.

## 2. Literature Review and Hypothetical Development

### 2.1. Leader Ethical Leadership and Employee OCB

Organizational citizenship behavior is defined as “contributions to the maintenance and enhancement of the social and psychological context that supports task performance” (Organ, 1997). It encompasses employees’ voluntary, discretionary, and altruistic activities beyond their job requirements (Podsakoff et al., 2000). It consists of five OCB components (Fassina et al., 2008): (1) altruism—voluntarily and selflessly assisting other employees in the organization without expecting anything in return, such as helping a new employee adapt to a new work environment; (2) conscientiousness—demonstrating proactive behavior beyond formal company rules, such as safeguarding organizational resources; (3) courtesy—informing colleagues of potential issues in advance to help them avoid workplace difficulties; (4) civic virtue—demonstrating a positive attitude and sense of responsibility towards company activities, such as actively participating in meetings; (5) sportsmanship—maintaining a positive attitude by refraining from behaviors such as complaints about minor inconveniences and actively addressing workplace challenges. These components are divided into two categories: OCBI, behaviors directed at other individuals to benefit them, and OCBO, behavior directed toward the organization to contribute to and benefit the organization (Williams & Anderson, 1991).

Previous studies have identified various antecedents that enhance employee OCB, including organizational identification (Sidorenkov et al., 2023), organizational commitment (Organ, 1990), job satisfaction (Zeinabadi, 2010), supportive organizational environment (Han et al., 2019), transformational leadership (Nohe & Hertel, 2017), leader–member exchange (Asgari et al., 2008), and role clarity and task importance (Piccolo & Colquitt, 2006). Beyond these organizational, task, and leadership characteristics, certain employee traits, such as emotional stability (Golafshani & Rahro, 2013), conscientiousness (Lapierre & Hackett, 2007), agreeableness (Ilies et al., 2009), and intrinsic and extrinsic motivation (Finkelstein, 2011), also promote OCB.

Conversely, stress (Soo & Ali, 2016) and work–family conflict (Yu et al., 2018) stifle OCB by depleting employees’ emotional and time resources, making it difficult to engage in discretionary efforts beyond job requirements. OCB is crucial due to its numerous benefits for both individuals and organizations. It enhances organizational performance and efficiency (Kumari & Thapliyal, 2017), strengthens team cohesion, and promotes team performance (Lin & Peng, 2010). At the individual level, OCB enhances employee well-being and job satisfaction (Davila & Finkelstein, 2013; Lestari & Ghaby, 2018), improves performance and performance evaluations (Allen & Rush, 1998), and fosters workplace relationships and cooperation (Podsakoff et al., 1997).

Ethical leadership is “the demonstration of normatively appropriate conduct through personal actions and interpersonal relationships, and the promotion of such conduct to followers through two-way communication, reinforcement, and decision-making” (Brown et al., 2005). Ethical leadership depends on two social learning pillars: the moral person and the moral manager. Ethical leaders are considered moral because they are honest and trustworthy, prioritize employee well-being, and uphold ethical standards in both their personal and professional lives (Brown & Trevino, 2006). They make decisions based on values and ethical decision rules, uphold fairness, and consider stakeholders’ interests and long-term outcomes (Babalola et al., 2016; Walumbwa et al., 2017). As moral managers, ethical leaders use their authority to advance ethics in the workplace. They establish and communicate ethical standards to subordinates, reinforcing compliance through rewards and corrective actions (Brown & Mitchell, 2010). Ethical leaders exemplify appropriate conduct through personal behavior and interpersonal relationships, fostering ethical behavior in others through communication, reinforcement, and decision making (Brown & Trevino, 2006; Moore et al., 2019).

Ethical leadership positively influences follower outcomes, including job satisfaction (Qing et al., 2020), employee well-being at work (Kalshoven & Boon, 2012), work engagement (Cheng et al., 2014), affective organizational commitment (Demirtas & Akdogan, 2015), voice behavior (Chen & Hou, 2016), in-role job performance, and extra-role performance (Zulqarnain et al., 2024). It also fosters ethical behavior among followers (Al Halbusi et al., 2021; Al Halbusi et al., 2023). Additionally, ethical leadership reduces organizational deviance (Neves & Story, 2015) and unethical behavior among employees (Mayer et al., 2010).

Previous research indicates that ethical leaders’ moral values affect personal moral intentions, which, in turn, affect OCB (O’Keefe et al., 2018). Arshad et al. (2021) found that fostering ethical leadership and leader–member exchanges encourages employee OCB. Ethical leadership fosters employees’ sense of moral identity, motivating them to engage in ethical behaviors such as OCB (Gerpott et al., 2019). Tan et al. (2019) found that ethical leadership is significantly and positively correlated with perceived organizational support, which, in turn, fosters OCB. Ethical leaders who show concern for their subordinates, prioritize their interests, instill meaning in work, and treat employees with dignity and respect increase the likelihood of employees contributing to the organization (Shareef & Atan, 2019). Although these studies demonstrate that ethical leadership positively influences prosocial behavior (such as OCB), they primarily focused on the individual level. This study moves beyond perceptual analyses to contextualize leadership research, exploring the positive impact of group-level ethical leadership on subordinates’ OCB. Accordingly, we propose the following hypothesis:

**Hypothesis 1.**  *Ethical Leadership positively impacts employee OCB*.

### 2.2. The Mediating Role of Team Ethical Climate

Ethical climate is “the shared perceptions of what is ethically correct behavior and how ethical issues should be handled” (Victor & Cullen, 1987). In other words, the ethical climate reflects shared values regarding acceptable and unacceptable behavior on ethical issues and provides employees with guidelines for resolving ethical dilemmas (Teng et al., 2020).

Ethical leadership theory assumes that ethical leaders act as moral managers who establish and implement reward and punishment systems to encourage and uphold ethical behaviors (Brown et al., 2005). They rely on reward systems to discipline subordinates who violate ethical standards and reward those who demonstrate ethical behavior. Through this process, teams develop a shared ethical consensus (Kuenzi et al., 2020). Prior studies indicate that ethical leaders’ daily actions significantly influence an organization’s ethical climate (Stringer, 2002). Aloustani et al. (2020) found a significant relationship between ethical leadership and ethical climate. The moral manager formally discusses ethics and ethical values, explaining how they guide important team decisions and actions. Therefore, this study posits a positive relationship between ethical leadership and ethical climate.

Ethical climate encompasses both an environmental influence that shapes employees’ behavior through reinforcement and a signal that reflects a team’s ethical character (Cullen et al., 2003; Mulki et al., 2009). This study conceptualizes ethical climate as (1) an environmental stimulus and (2) a reflection of team-level social learning. Through external reinforcement, a collective ethical perception significantly influences employees’ attitudes and behaviors within team-level social learning. When immersed in an ethical environment, employees learn and exhibit ethical behaviors by aligning with organizational practices. They also observe, experience, and interpret various ethical actions within the organization (Teng et al., 2020).

Teams with a strong ethical climate often have formal, externally reinforced systems that either reward ethical behavior or penalize unethical behavior. These tangible external rewards enhance employees’ motivation for prosocial behavior (Bai et al., 2019). Similarly, team members in an ethically strong environment may also be rewarded for their ethical conduct or disciplined for unethical behavior. As a result, team members learn to align their behavior with the team’s ethical climate. For example, they are more likely to engage in prosocial behaviors such as OCB (Aloustani et al., 2020).

Accordingly, this study suggests that team members who respond to similar social cues in a shared ethical climate are more likely to engage in OCB. Previous studies have demonstrated that ethical leadership strengthens ethical climate and ethical climate positively impacts employee OCB, respectively. However, the studies have not considered ethical climate as a potential group-level mediating mechanism between ethical leadership and OCB. Based on this logic, this study posits that the group ethical climate acts as a team-level learning pathway between ethical leadership and employees’ positive behavior (OCB). Therefore, we formulate the following hypotheses:

**Hypothesis 2.**  *Ethical Leadership positively influences the team’s ethical climate*.

**Hypothesis 3.**  *The team’s ethical climate mediates the relationship between ethical leadership and employee OCB*.

### 2.3. The Mediating Role of Ethical Role Modeling

Ethical role model perception is defined as “the extent to which employees perceive the supervisor to be a personal role model when dealing with ethical dilemmas and decision-making” (Ogunfowora, 2013). Ethical leaders are typically trustworthy, reliable, honest, and caring (Brown & Trevino, 2006). They foster fair work environments and address employee needs. Through such behaviors, ethical leaders are generally regarded as ethical role models.

Brown et al. (2005) emphasized the significance of role modeling in ethical leadership’s social learning process. Subordinates observe, interpret, and assess a leader’s role modeling, ultimately adopting similar actions. The individual’s social learning process consists of two sub-processes—attention and retention—that shape subordinates’ cognitive development over time. The first sub-process, attention, ensures that subordinates focus on both the role model and the role model’s behavior (Wood & Bandura, 1989). Ethical leaders exhibit characteristics aligned with fundamental ethical principles, which followers perceive as “normatively appropriate and motivated by altruism” (Brown et al., 2005).

The second sub-process, retention, involves subordinates cognitively encoding a role model’s behavior into a form that is easily retained in memory (Bai et al., 2019). Ethical leaders intentionally communicate ethical values and establish rewards and sanctions to reinforce ethical standards and regulate followers’ behaviors. Such managerial actions are theorized to strengthen followers’ perceptions of the leader as an ethical role model (Ko et al., 2018). According to social learning theory, whether an individual is perceived as a role model depends on their power and status (Bandura, 1977; Manz & Sims, 1986). That is, leaders should hold formal managerial positions and possess the authority to reward (punish) ethical (unethical) conduct. Thus, a leader’s ethical actions should be salient to followers, reinforcing their perception of the leader as an ethical role model (Wang et al., 2019).

According to social learning theory (Bandura, 1977), individuals primarily learn social behaviors by observing role models. Therefore, when subordinates perceive a leader as an ethical role model, they develop a clearer understanding of expected normative behaviors and are more likely to emulate the leader’s ethical conduct (Ogunfowora, 2014). Role models, in effect, shape individuals’ self-identity and guide their actions. Modeling is a powerful mechanism for learning ethical actions in the workplace (Weaver et al., 2005). Prior research indicates that the perception of ethical role modeling mediates the relationship between ethical leadership and unethical behavior. Ethical leadership reinforces social learning by shaping followers’ self-perception as role models, thereby reducing unethical behavior among subordinates (Wang et al., 2019). Ethical role modeling mediates the relationship between perceived ethical leadership and employee promotive and prohibitive voice behaviors, a form of OCB (Bai et al., 2019). However, the existing literature has not directly explored the impact of ethical role modeling on employee OCB. Therefore, from the “moral person” perspective of moral leadership, this study proposes that ethical role modeling enables employees to learn from ethical leaders and demonstrate greater OCB. Accordingly, we propose the following hypotheses:

**Hypothesis 4.**  *Ethical Leadership positively influences ethical role modeling*.

**Hypothesis 5.**  *Ethical role modeling mediates the relationship between ethical leadership and employee OCB*.

### 2.4. The Mediating Role of Affective Well-Being

Job-related affective well-being is assessed based on positive and negative feelings experienced in the workplace (Warr, 1987). Warr (1990) categorized work-related positive and negative emotions using a two-dimensional scale based on pleasure and arousal. The scale includes six positive emotions (i.e., optimism, cheerfulness, calm, content, enthusiasm, and relaxation) and six negative emotions (i.e., depressed, tense, uneasy, worried, gloomy, and miserable).

Conservation of resources (COR) theory (Hobfoll, 1989) posits that individuals are motivated to obtain, retain, and protect resources they consider valuable. Resources are defined as “objects, personal characteristics, conditions, or energies that are valuable in themselves or because they act as conduits for the achievements or protection of valued resources”. According to COR theory, resources, such as ethical leadership, enable employees to acquire more resources. This positive cycle of resources can positively impact well-being (Kaffashpoor & Sadeghian, 2020).

When a supervisor respects an employee’s rights and dignity, prioritizes their welfare, and attentively listens to their concerns and ideas, the employee is likely to experience positive emotions such as happiness or optimism (Brown & Mitchell, 2010; Avey et al., 2012). Conversely, employees may experience negative emotions such as unhappiness, anger, and worry if they perceive their leader as unethical, for instance, failing to address unethical behavior or neglecting to communicate ethical expectations and mutual respect (Tan et al., 2019). Ethical leadership is characterized by key traits, such as honesty, trustworthiness, justice, conscientiousness, and compassion. With these traits, leaders encourage employees to express concerns and make fair decisions on important matters (Hendriks et al., 2020). As a result, subordinates receive support, attention, and emotional care from their leader, positively influencing their well-being (Ahmad et al., 2018). Therefore, ethical leaders enhance followers’ affective well-being by fostering a conducive work environment, which results in positive emotional experiences (Arshad et al., 2021). Ahmad et al. (2018) found that ethical leaders’ integrity, consideration, and support can enhance subordinates’ positive emotions while reducing negative feelings and stress in an academic work environment.

COR theory (Hobfoll, 1989) is particularly valuable for understanding the impact of affective well-being on OCB. While negative emotions deplete employees’ resources, strong affective well-being enables individuals to preserve and expand personal and work resources. Xu et al. (2019) found that employees with strong affective well-being set more ambitious goals, which motivate them to generate additional work resources to achieve these goals. Therefore, this study proposes that affective well-being enhances employees’ engagement in organizational citizenship behaviors, which in turn help them build work resources such as strong interpersonal relationships. Employees who engage in organizational citizenship behavior invest personal and work resources in activities beyond their formal job requirements (Koopman et al., 2016). When employees take on additional responsibilities beyond their job requirements, they may require more time and energy to fulfill their obligations. For instance, assisting colleagues with problem solving can increase their personal workload.

Previous research indicates that OCB may require significant human and financial resources and can lead to employee exhaustion (Bergeron et al., 2014). Organizational citizenship behavior is time-consuming and can hinder employees’ work progress (Bolino et al., 2013). Therefore, high-level affective well-being serves as a resource that enables employees to engage in OCB. Hobfoll (2001) argues that “people must invest resources to prevent resource loss, recover from the loss, and obtain resources.” Therefore, the acquired resources are often reinvested in the organization.

Accordingly, this study proposes that employees with strong affective well-being are more likely to demonstrate OCB as a means of reciprocating the resources they have obtained (Xu et al., 2019). In previous studies, well-being was considered both an antecedent and an outcome of OCB. Drawing on the COR theory, this study positions well-being as an antecedent of OCB to establish a link between ethical leadership and OCB, providing further empirical evidence for the causal relationship. Thus, we propose the following hypotheses:

**Hypothesis 6.**  *Ethical Leadership positively influences affective well-being*.

**Hypothesis 7.**  *Affective well-being mediates the relationship between ethical leadership and employee OCB*.

**Figure 1:**
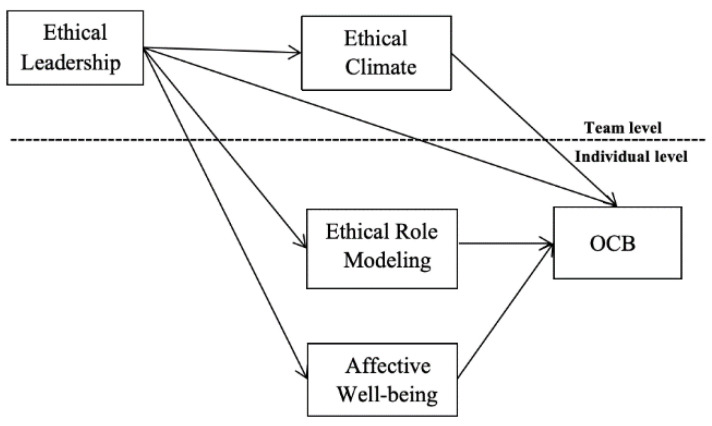
A multilevel mediation model.

The research model is shown in Figure 1 below.

## 3. Method

### 3.1. Sample and Procedure

Given that this study focuses on SMEs, we obtained a list of member companies from the official website of the China Association of Small and Medium Enterprises. From this list, we selected SMEs from Guangdong Province, which has the highest number of SMEs in China. For a company to be selected, it must have an official website with contact information available. Using the contact details provided on the website, we contacted each company’s human resources department. After obtaining approval from the team leader, the human resources department shared the team leader’s contact information. When distributing questionnaires, different team numbers were assigned to each team leader. To ensure respondents could answer the questionnaire’s “Please write down your team number” question, we requested that team leaders inform their members of their respective team numbers.

All participants received a paper questionnaire and a return envelope, which they used to return the completed questionnaire. At the beginning of the questionnaire, we assured each participant of anonymity and confidentiality and outlined the purpose of the study. The participants completed the questionnaire after reading and agreeing to our confidentiality statement. Because all participants were Chinese, we implemented the back-translation procedure proposed by Brislin (1980). First, two bilingual researchers, certified English translators, independently translated all original measurement items into Chinese and back-translated them into English to ensure semantic equivalence and agreement. A total of 512 questionnaires were collected from 85 teams. After excluding invalid or incomplete questionnaires, 426 valid questionnaires from 71 teams were collected. Each group consists of a leader and six members.

Descriptive statistics on the demographic characteristics of the valid sample were conducted using SPSS 26.0. Among the 71 supervisors, 64.79% (N = 46) were male, and 35.21% (N = 25) were female. The largest age group among supervisors was 31–40 years old, constituting 52.11% (N = 37) of the sample, followed by 41–50 years old (26.76%, N = 19) and 20–30 years old (21.13%, N = 15). In terms of supervisors’ work experience, 29.58% (N = 21) had 1–3 years of experience, 43.66% (N = 31) had 4–6 years, and 26.76% (N = 19) had 7–10 years. Regarding supervisors’ educational level, 12.68% (N = 9) had a high school diploma or lower, 22.53% (N = 16) had a college degree, and 64.79% (N = 46) had a bachelor’s degree.

Among the 426 subordinates, 56.57% (N = 241) were male, and 43.43% (N = 185) were female. Most of the subordinates belonged to the 31–40 age group, comprising 55.87% (N = 238) of the sample, followed by the 20–30 age group (N = 179, 42.02%) and the 41–50 age group (N = 9, 2.11%). In terms of subordinates’ work experience, 78.64% (N = 335) had 1–3 years of experience, 17.37% (N = 74) had 4–6 years, and 3.99% (N = 17) had 7–10 years. Regarding subordinates’ educational levels, 1.7% (N = 33) had a high school diploma or lower, 15.1% (N = 95) had a college degree, and 83.2% (N = 298) had a bachelor’s degree.

### 3.2. Measures

The 10-item ethical leadership scale developed by Brown et al. (2005) was adopted to measure leaders’ ethical behaviors. One sample item is: “My leader discusses business ethics or values with employees.” The ethical climate was assessed using Schwepker’s (2001) 7-item scale. A sample item is: “My work team has a formal, written code of ethics”. Three items developed by Ogunfowora (2013) were used to measure ethical role modeling. A sample item is: “When faced with an ethical dilemma at work, I usually follow the examples set by my leader in the past.” Participants were asked to respond to the statements above using a 5-point Likert-type scale, ranging from 1 = strongly disagree to 5 = strongly agree.

Job-related affective well-being was assessed using the 12-item scale developed by Warr (1990). Employees were asked, “Thinking of the past few weeks, how often has your job made you feel each of the following: cheerful, enthusiastic, relaxed, optimistic, calm, contented, gloomy, worried, depressed, tense, miserable, and uneasy.” Participants used a 5-point Likert scale to respond to the negative feeling statements, ranging from 1 = always to 5 = never, and to the positive feeling statements, ranging from 1 = never to 5 = always. OCB was assessed using Lee and Allen’s 16-item scale (Lee & Allen, 2002). One sample item is “Give up time to help others who have work or nonwork problems.” Participants used a 5-point Likert scale to respond to the statements, ranging from 1 = strongly disagree to 5 = strongly agree.

### 3.3. Analysis Strategy

Subordinates were nested within workgroups supervised by managers. To account for the impact of the supervisor’s ethical leadership on subordinate-level outcomes, we adopted a multilevel approach to address the hierarchical structure in which subordinate responses were nested within the supervisors’ workgroups. Therefore, a group leader’s ethical leadership and the group’s ethical climate were level-2 variables, and ethical role modeling, affective well-being, and organizational citizenship behavior were level-1 variables. Given the nested nature of the data (i.e., 426 employee-leader dyads nested within 71 groups) and the presence of multilevel effects, we conducted a multilevel analysis to test the proposed models.

We aggregated employees’ perceptions of ethical leadership and ethical climate by estimating the cluster mean to measure team ethical leadership and team ethical climate (Glick, 1985). To justify the aggregation, we calculated intraclass correlations (*ICC1*), the reliability of mean group score (*ICC2*; Bliese, 2000), and within-group agreement (*rwg*; James et al., 1993). The *rwg* for team ethical leadership and team ethical climate in this study were 0.813 and 0.826, respectively, exceeding the 0.70 threshold, indicating strong within-group agreement. The *ICC1* values of ethical leadership and ethical climate were 0.415 and 0.433. The *ICC2* values of ethical leadership and ethical climate were 0.810 and 0.821, confirming the reliability of the group means within the sample. These results indicate that conceptualizing and analyzing ethical leadership and ethical climate at the team level were statistically appropriate.

According to Zhang et al. (2009), the mediating role of team ethical climate on the relationship between ethical leadership and employee OCB was classified as a 2-2-1 model, the cross-level mediation effect-higher mediator. Similarly, the mediation of ethical role modeling and affective well-being in the relationship between ethical leadership and employee OCB was categorized as a 2-1-1 model, representing a lower-level cross-level mediation effect.

We conducted a confirmatory factor analysis using MPlus 8.3 to assess the model’s fit. Model fit was evaluated using the following goodness-of-fit criteria (Browne & Cudeck, 1992): the standardized root-mean-square residual (SRMR) and the root-mean-square error of approximation (RMSEA) should be <0.08, the Tucker–Lewis fit index (TLI) and comparative fit index (CFI) should be >0.9, and the chi-squared goodness-of-fit divided by degrees of freedom (χ^2^/df) should be <3. The results showed that χ^2^/DF = 1.044 (<3), CFI = 0.996 (>0.9), TLI = 0.994 (>0.9), RMSEA = 0.026 (<0.08), and SRMR = 0.042 (<0.08).

## 4. Results

### 4.1. Preliminary Analyses

The reliability and validity of questionnaires were first tested. As shown in Table 1, all Cronbach’s alpha values were greater than 0.70 (George & Mallery, 2003), verifying the internal consistency of all variables. Additionally, all CR values exceeded 0.70, and all average variance extracted (AVE) values exceeded 0.50 (Hair et al., 1998), indicating that the reliability and validity scores of all constructs were acceptable.

Table 2 shows that all variables’ standard deviations fall within the normal range. The variables exhibit the expected binary correlation. Additionally, discriminant validity is demonstrated by the square roots of the AVE values shown on the diagonal line exceeding the correlation values (Fornell & Larcker, 1981). Consequently, the data were appropriate for further analysis.

### 4.2. Hypothesis Tests

Table 3 reports the coefficients of all paths in the multilevel model between ethical leadership and employee OCB. Hypothesis 1 predicts that the team leader’s ethical leadership would be positively related to employee OCB. The regression coefficient for the relationship between team-level ethical leadership and employee OCB is 0.436 (*p* < 0.001). Thus, a one-point increase in a team leader’s ethical leadership is associated with a 0.436-point increase in employee OCB. These results support Hypothesis 1. Hypotheses 2, 4, and 6 predict a positive relationship between the team leader’s ethical leadership and ethical climate, ethical role modeling, and affective well-being. The analysis results show that the team leader’s ethical leadership has significant positive effects on team ethical climate (β = 0.412, *p* < 0.001), ethical role modeling (β = 0.447, *p* < 0.001), and affective well-being (β = 0.364, *p* < 0.001). Therefore, Hypotheses 2, 4, and 6 are all supported.

Furthermore, team ethical climate (β = 0.216, *p* < 0.001), ethical role modeling (β = 0.495, *p* < 0.001), and affective well-being (β = 0.206, *p* < 0.001) all have significant positive effects on OCB.

Table 4 summarizes the multilevel mediation effects between the supervisor’s ethical leadership and employee OCB. The findings show a significant positive mediation effect (ethical leadership → ethical climate → OCB) of 0.089 (0.412 × 0.216, *p* < 0.01), which is statistically significant. Thus, Hypothesis 3 is supported. The results also reveal a significant positive mediation effect (ethical leadership → ethical role modeling → OCB) of 0.221 (0.447 × 0.495, *p* < 0.001). Therefore, Hypothesis 5 is supported. The findings further indicate a significant positive mediation effect (ethical leadership → affective well-being → OCB) of 0.075 (0.364 × 0.206), *p* < 0.01. Therefore, Hypothesis 7 is supported. Furthermore, the bootstrap 95% confidence intervals corresponding to the indirect effects of ethical leadership on OCB through ethical climate (0.052–0.161), ethical role modeling (0.146–0.283), and affective well-being (0.051–0.149) do not contain 0, indicating that the mediating effects of each variable are statistically significant.

## 5. Discussion

This study employed a multilevel mediation model for small and medium enterprises in China to examine the influence of team-level ethical leadership on individual-level organizational citizenship behavior. It identifies the mediating effect of team-level ethical climate, individual-level ethical role modeling, and individual-level affective well-being in the relationship between a team leader’s ethical leadership and employee OCB. Additionally, it establishes the underlying influence mechanism of ethical leadership. Below is a summary of the study’s primary conclusions based on the empirical findings.

First, ethical leadership at the team level significantly improves employee organizational citizenship behavior at the individual level. Second, team-level ethical leadership has a significant positive impact on team-level ethical climate, individual-level ethical role modeling, and individual-level affective well-being. Third, team-level ethical climate, individual-level ethical role modeling, and individual-level affective well-being partially mediate the relationship between a leader’s team-level ethical leadership and individual-level OCB.

### 5.1. Theoretical Implications

This study has several significant theoretical implications. First, this study finds that team ethical leadership has a significant positive effect on employee OCB, consistent with Aloustani et al. (2020). However, most studies focus on a single level of analysis; there is an increasing demand for existing organizational theories to be ‘more context-sensitive’ (Bai et al., 2019). To address this need, this study establishes a multilevel model to investigate the connection between a leader’s ethical leadership and employee OCB. Additionally, this study shifts the focus of current research from viewing OCB as a result of individual-level factors (Saleem et al., 2017; Uzun, 2018) to emphasizing the importance of employees observing and learning from their surrounding environment and role models. This shift expands the application of social learning theory by highlighting its relevance to the two pillars of ethical leadership.

Second, this study finds that team ethical climate has a significant mediating effect on the relationship between ethical leadership and subordinates’ organizational citizenship behavior. In previous research, various benefits of an ethical work climate have been identified, including employee job satisfaction, organizational commitment (Asgari et al., 2019; Teresi et al., 2019), reduced work role stress, lower turnover intention, and improved job performance (Jaramillo et al., 2006; Mehmood et al., 2018). These studies have shown that ethical climate has a positive impact on employees’ in-role attitudes and behavioral outcomes. However, few studies have focused on whether ethical climate also affects employees’ extra-role attitudes and behaviors (Teng et al., 2020).

Therefore, this study introduces team ethical climate as a mediating mechanism between ethical leadership and OCB. When leaders demonstrate ethical leadership, they can foster an atmosphere where doing the right thing is valued (Brown et al., 2005). An ethical environment with high ethical standards and strong morale consistently reinforces the importance of making ethical decisions and taking ethical actions. This message guides employees to perform their jobs ethically and engage in extra-role behaviors, such as OCB (Teng et al., 2020). This result supports empirical evidence on the application of social learning theory from a group perspective. When employees share a common understanding of ethical behavior within their team, those who learn to act ethically in such a climate are more likely to commit to the organization and engage in more OCB.

Third, this study directly tests the often-theorized role modeling process, which has been proposed as the basis for the relationship between ethical leadership and subordinates’ OCB. Previous studies have found that ethical leadership fosters moral role modeling, which is consistent with the results of this study. The main way that people learn how to act in social settings is by observing models or examples. Ethical leaders act as role models for subordinates and influence their ethical or unethical behavior through social learning processes (Brown & Trevino, 2006). Although widely recognized as a mechanism, this process has lacked empirical testing (Bai et al., 2019). The current study supports social learning theory by demonstrating the mediating effect of ethical role modeling. Notably, this study also finds that subordinates’ ethical role modeling was more strongly associated with subordinates’ organizational citizenship behavior than ethical leadership. This result aligns with recent research showing that ethical role modeling is closer to subordinates’ behavior. Moreover, the extent to which followers emulate a leader’s behavior depends on their perception of the leader as a role model rather than solely on their perception of the leader’s behavior (Wang et al., 2019).

Fourth, with the development of positive psychology, employee well-being is becoming a central topic (He et al., 2019). Previous studies have shown that employee well-being can effectively promote employee OCB (Xu et al., 2019), which is consistent with the empirical results of this study. This study further examined how affective well-being significantly mediates the relationship between ethical leadership and employee OCB. Based on COR theory, ethical leadership serves as a resource for subordinates by demonstrating care, fairness, and attentiveness to employees’ opinions. This support enhances subordinates’ affective well-being, which, in turn, motivates them to invest their excess resources back into the organization by engaging in OCB. This study’s results provide empirical support for the application of COR theory in ethical leadership and positive psychology.

### 5.2. Practical Implications

This study explored the value of group-level ethical leadership in motivating and predicting employees’ organizational citizenship behavior. As suggested by the model, a key issue for leaders is fostering a consistent ethical climate, serving as role models and improving employees’ affective well-being. The results suggest several practical implications.

First, to help leaders understand the importance of ethical leadership and demonstrate stronger ethical leadership, organizations should evaluate, develop, and reward ethical leadership behaviors. Organizations can also establish training programs on ethical dilemmas and decision making to help leaders improve behaviors that conform to moral standards (Bai et al., 2019). Second, HR departments should exercise caution when selecting team leaders; selection procedures should include assessments of past leadership performance and attitudes on ethical issues. Organizations may adopt various selection methods, such as assessments of integrity and moral standards (Wang et al., 2019).

Third, compared with large companies, small and medium companies lack formal rules and regulations (Kararti & Yuksekbilgili, 2014). Given the effective mediating role of team ethical climate between a leader’s ethical leadership and employee OCB, each workgroup should have a clearly written ethics policy that outlines employee expectations and specifies what is and is not considered acceptable. This will allow all team members to develop shared ethical values (Dinc & Aydemir, 2014). Employees’ ethical consciousness and the group’s ethical climate will improve as a result.

Fourth, this study’s results indicate that employees who experience high levels of affective well-being are more likely to engage in organizational citizenship behavior. According to research by Kalshoven and Boon (2012), employees with low workplace well-being may be less productive, absent more frequently, and likely to make lower-quality decisions. Therefore, it is important for organizations to focus on improving employee well-being (Akram Ahmad & Obeid Al-Shbiel, 2019). According to the findings of this study, organizations can improve employee well-being by using ethical leadership practices, including fairness, caring, and power sharing.

### 5.3. Research Limitations and Future Directions

Although this study is founded on classic theories and provides statistical support for its hypotheses, it has certain limitations. First, it employs a cross-sectional research approach. For instance, even though this study demonstrates that employee OCB is encouraged by ethical climate and affective well-being, it is possible that employee OCB, in turn, improves the team’s ethical climate (Bai et al., 2019) and enhances their well-being (Johansson & Hart, 2023). Future research should use a longitudinal design and focus on the directionality of the causal relationship between the variables examined in this study.

Second, the data were collected through a self-report survey instrument. It is possible that respondents’ self-reported responses may not have reflected their true circumstances (Siemsen et al., 2010). A more reliable approach would be to have leaders evaluate employee OCB.

Third, although this study demonstrates the significant influence of ethical leadership on ethical role modeling, the assessment of role modeling may also include non-supervisory role models. Given that previous research suggests coworkers may influence certain organizational behaviors more strongly than leaders, future research should also consider their role in the workplace. O’Keefe et al. (2018) found that respondents who perceived their coworkers as highly ethical were more likely to behave ethically. Therefore, in addition to leaders’ influence on employees’ ethical role modeling, colleagues may also shape ethical role modeling.

Fourth, in addition to a positive construct, future research could consider using counterproductive work behavior (CWB) as an outcome variable to study the validity of the antecedents in this model, that is, whether they effectively reduce negative outcomes in the workplace.

Finally, because Chinese society is based on social hierarchies, subordinates may be more influenced by their leaders because of differences in authority, power, and social standing. This dynamic makes them more inclined to view the leader as a role model (Lian et al., 2012). In cultures with low power distance, will leaders’ influence as role models weaken? Moreover, Chinese society is collectivist, and individuals are more likely to be affected by a collective climate. In individualistic cultures, the influence of organizational climate on employees may also be weakened. Since this study focused on a single culture (China), more research is needed to generalize the findings to other cultures. Future studies should explore potential cross-cultural differences (Wang et al., 2019). These cultural differences may affect the strength of the relationship between ethical leadership, ethical climate, ethical role modeling, and organizational citizenship behavior. In addition, this study focuses on small- and medium-sized enterprises. Future research can apply the model to large enterprises to examine whether the relationship between variables changes across organizations of different sizes.

## Figures and Tables

**Table 1:** Scale reliability and validity.

Variable	Items	Cronbach’s Alpha	Factor Loading	CR	AVE
Ethical leadership	10	0.889	0.653–0.844	0.925	0.544
Ethical role modeling	3	0.869	0.776–0.791	0.896	0.686
Ethical climate	7	0.887	0.630–0.916	0.920	0.597
Affective well-being	12	0.862	0.624–0.889	0.903	0.531
OCB	16	0.946	0.723–0.912	0.956	0.728

AVE = average variance extracted, CR = composite reliability.

**Table 2:** Correlation matrix of the variables.

Variable	Mean	SD	1	2	3	4	5
1. Ethical leadership	4.063	0.439	(0.737)				
2. Ethical role modeling	3.956	0.701	0.465 **	(0.828)			
3. Ethical climate	4.020	0.408	0.309 **	0.549 **	(0.773)		
4. Affective well-being	3.828	0.485	0.369 **	0.482 **	0.355 **	(0.729)	
5. OCB	4.145	0.612	0.439 **	0.373 **	0.324 **	0.237 **	(0.853)

**: The Pearson correlation coefficient is significant at a 0.01 level; the square root of AVE is presented along the diagonal.

**Table 3:** Path coefficients of the multilevel model between ethical leadership and OCB.

	Estimates	95%CI	Remarks
**Team → Team**			
Ethical Leadership → Ethical Climate	0.412 ***	(0.355, 0.492)	Supported (H2)
**Team → Individual**			
Ethical Leadership → OCB	0.436 ***	(0.374, 0.522)	Supported (H1)
Ethical Leadership → Ethical Role Modeling	0.447 ***	(0.393, 0.565)	Supported (H4)
Ethical Leadership → Affective Well-being	0.364 ***	(0.304, 0.444)	Supported (H6)
Ethical Climate → OCB	0.216 ***	(0.131, 0.292)	
**Individual → Individual**			
Ethical Role Modeling → OCB	0.495 ***	(0.449, 0.571)	
Affective Well-being → OCB	0.206 ***	(0.136, 0.276)	

*** *p* < 0.001. Bootstrap sample = 1000. CI = confidence interval.

**Table 4:** Summary of multilevel mediation impacts.

	Estimates	95%CI	Remarks
**Team→ Team → Individual**			
Ethical Leadership → Ethical Climate→ OCB	0.089 **	(0.052, 0.161)	Supported (H3)
**Team → Individual → Individual**			
Ethical Leadership → Ethical Role Modeling → OCB	0.221 ***	(0.146, 0.283)	Supported (H5)
**Team → Individual → Individual**			
Ethical Leadership →Affective	0.075 **	(0.051, 0.149)	Supported (H7)
Wellbeing → OCB			

*** *p* < 0.001; ** *p* < 0.01.

## Data Availability

Data will be made available upon request.
